# Autopsy findings of pediatric COVID-19: a systematic review

**DOI:** 10.1186/s41935-022-00288-0

**Published:** 2022-07-14

**Authors:** Gilbert Sterling Octavius, Jeremiah Hilkiah Wijaya, Alexa Ovilia Tan, Michelle Patricia Muljono, Shally Chandra, Andry Juliansen

**Affiliations:** Department of Pediatrics, Faculty of Medicine, Universitas Pelita Harapan, MH Thmarin Boulevard 1100, Karawaci, Tangerang, Banten 15811 Indonesia

**Keywords:** Autopsy, Pathology, COVID-19, Pediatric, Pathophysiological mechanisms

## Abstract

**Background:**

Little is known how COVID-19 is affecting children. Autopsies help gain an understanding of the pathophysiology of new and developing diseases. Numerous post-mortem studies had been conducted in adults with COVID-19, but few in children. Thereby, this systematic review aims to investigate the autopsy findings from pediatric COVID-19 patients.

**Results:**

There were a total of 15 patients from eight studies. COVID-19 mainly affects the heart and lungs. Pathology findings from the heart of COVID-19 pediatric patients include diffuse inflammatory infiltrate, myocarditis, cardiomyocyte necrosis, pericarditis, and interstitial edema. Histopathology abnormalities observed in the lungs are diffuse alveolar damage, cytopathic changes, thrombi in arterioles and septal capillaries, lung congestion, focal acute hemorrhage and edema, focal exudative changes, and mild pneumocyte hyperplasia. In addition, pathological findings from other organs, such as the liver, kidney, brain, bone marrow, lymph node, skin, spleen, muscle, colon, parotid gland, and adrenal of COVID-19 pediatric patients are also included in this review.

**Conclusion:**

Cardiomyocyte necrosis, interstitial edema, lung congestion, and diffuse alveolar damage are the most significant pathologic findings of the heart and lung in pediatric COVID-19 patients. More studies are needed to elucidate the pathophysiology of SARS-CoV-2 in autopsy findings and to determine the exact cause of death since it could be related to COVID-19 or other comorbidities.

## Background

At the start of the pandemic, little is known how COVID-19 is affecting children. It seems that children are somewhat protected from COVID-19 due to lower exposure to the outside world, reduced number of angiotension converting enzyme 2 (ACE2) receptors in children, and the fact that children have a strong innate immune response due to trained immunity (Patel and Verma 2020; Dhochak et al. 2020). However, for the last few months, children’s cases have been on the rise in several nations worldwide due to the current pandemic, such as in Indonesia (72,762 cases per 21 December 2020) and China (2135 cases per 8 February 2021) (Dong et al. 2020). The rise of children’s COVID-19 cases is underlined by several factors, including school reopening, daycare, unavailability of COVID-19 vaccination, the new variants of the virus, and others (Jenco 2021). Although children have a much lower overall mortality rate compared to adults; they are at risk of suffering from the multisystem inflammatory syndrome in children (MIS-C) that contribute to the increasing death due to COVID-19 (Hoste et al. 2021).

Autopsies reports help reveal the pathophysiology of new and developing diseases. Autopsies performed during earlier coronavirus pandemics, such as severe acute respiratory syndrome (SARS) caused by SARS coronavirus 1 (SARS-CoV-1) in 2002 and Middle East Respiratory Syndrome (MERS) caused by MERS-related coronavirus (MERS-CoV) in 2012, revealed insights into their pathophysiology and helped improve patient management (Hwang et al. 2005; Ng et al. 2016). The new COVID-19 disease has infected almost every country in the world (Weekly epidemiological update on COVID-19 - 8 February 2022 2022).

The COVID-19 pandemic is a rapidly growing body of research, with publications gradually unravelling the pathophysiology and underlying pathology (Fox et al. 2020). Numerous post-mortem studies had been conducted in adults but few only in children (Dell’Aquila et al. 2020; De Michele et al. 2020). Despite a strong push to do autopsies on COVID-19 patients, the overall autopsy rate appears to be low at the moment (Williamson 2020). To perform an autopsy on patients who died due to COVID-19 is vital to answer critical questions about COVID-19. Thereby, this systematic review aimed to investigate the autopsy findings from pediatric COVID-19 patients, especially the cause of deaths of each patient correlated with histopathology findings from each organ(s). As a secondary purpose, we also collected clinical data and laboratory data amongst pediatric patients to observe any trends in pediatric COVID-19 related mortality.

## Methods

In this systematic review, the preferred reporting item declared by the systematic review and meta-analysis (PRISMA) 2020 was followed (Page et al. 2021; Rethlefsen et al. 2021). The log of this systematic review has been uploaded to the PROSPERO database (CRD42021269497).

The literature search was limited from December 2019 to June 2021, which included all languages. All case series, case reports, cross-sectional studies, clinical trials, and cohort studies that report an autopsy in confirmed pediatric (aged 0–18 years old) COVID-19 patients, a multisystem inflammatory syndrome in children (MIS-C), and pediatric inflammatory multisystem syndrome-temporally associated with SARS-COV-2 (PIMS-TS) will be included in this review. Exclusion criteria comprised no autopsy related, molecular study, not SARS-COV-2-related, no pathology data, and animal studies. Abstracts, letters to the editor, and reviews were screened for references to ensure literature saturation before they were excluded.

The literature search started on 25 July 2021 and ended on 27 July 2021. The authors utilized eight databases, including PubMed, Science Direct, MEDLINE, Scielo, Medrxiv, Research Square, and Biorxiv, without restrictions on the language. Table 1 contains a list of keywords used in each database. The comprehensive data that was compiled were characteristic of patients demographic (age, race, sex, body mass index (BMI), comorbid disease, first symptom onset), post-mortem COVID-19 testing, lab parameter (hemoglobin, hematocrit, platelets, white blood cell count, lymphocytes, absolute neutrophil count, urea, creatinine, D-dimer, troponin, creatine kinase myocardial band, interleukin-6, creatine kinase, blood pH, bicarbonate, partial pressure of carbon dioxide (PaCO_2_), partial pressure of oxygen (PaO_2_), central venous oxygen saturation (ScvO_2_), lactate, C-reactive protein (CRP), erythrocyte sedimentation rate (ESR), total protein, albumin, total bilirubin, aspartate aminotransferase (AST), alanine aminotransferase (ALT), and gamma-glutamyl transferase (GGT). Postmortem examination would also be tabulated, including the heart, lung, nasopharyngeal polymerase chain reaction (PCR) test, cerebrospinal fluid (CSF) culture, and immune deficiency test. Data of post-mortem toxicology such as blood culture and vitreous examination are tabulated in this review. A separate email will be sent to the corresponding research author if the reviewer found some data missing from this systematic review.

**Table 1:** Keywords used in each database platform

Database	Keyword or medical subject headings
MEDLINE	(((COVID-19) AND MIS-C) AND Autopsy) AND Pediatric) AND Histopathology
Research Square	(COVID-19 (OR) SARS-COV-2 (OR) PIMS-TS (OR) MIS-C) AND (pediatric (OR) Adolescent) AND (Autopsy (OR) Pathology)
Google Scholar	(COVID-19 (OR) MIS-C (OR) PIMS-TS) AND (Pediatric (OR) Adolescent) AND (Autopsy" (OR) "Histopathology)
PubMed	"childs"[All Fields] OR ("paediatrics"[All Fields] OR "pediatrics"[MeSH Terms] OR "pediatrics"[All Fields] OR "paediatric"[All Fields] OR "pediatric"[All Fields]) OR ("infant"[MeSH Terms] OR "infant"[All Fields] OR "infants"[All Fields] OR "infant s"[All Fields]) OR ("infant, newborn"[MeSH Terms] OR ("infant"[All Fields] AND "newborn"[All Fields]) OR "newborn infant"[All Fields] OR "neonatal"[All Fields] OR "neonate"[All Fields] OR "neonates"[All Fields] OR "neonatality"[All Fields] OR "neonatals"[All Fields] OR "neonates"[All Fields]) OR ("adolescences"[All Fields] OR "adolescency"[All Fields] OR "adolescent"[MeSH Terms] OR "adolescent"[All Fields] OR "adolescence"[All Fields] OR "adolescents"[All Fields] OR "adolescent s"[All Fields])) AND ("autopsied"[All Fields] OR "autopsy"[MeSH Terms] OR "autopsy"[All Fields] OR "autopsies"[All Fields] OR ("pathology"[MeSH Terms] OR "pathology"[All Fields] OR "pathologies"[All Fields] OR "pathology"[MeSH Subheading]) OR ("histopathologies"[All Fields] OR "pathology"[MeSH Subheading] OR "pathology"[All Fields] OR "histopathology"[All Fields] OR "pathology"[MeSH Terms]) OR "post-mortem"[All Fields] OR "post-humous"[All Fields] OR ("autopsy"[MeSH Terms] OR "autopsy"[All Fields] OR "necropsy"[All Fields] OR "necropsied"[All Fields] OR "necropsies"[All Fields]))) AND (allchild[Filter])
Science Direct	(COVID-19 (OR) SARS-COV-2 (OR) PIMS-TS (OR) MIS-C) AND (pediatric (OR) Adolescent) AND (Autopsy (OR) Pathology)
Scielo	( (COVID-19) OR (SARS-COV-2) OR (PIMS-TS) OR (MIS-C) OR (Multisystem inflammatory syndrome in children) OR (COVID-2019)) AND ((pediatric) OR (children) OR (Infant) OR (Neonate) OR (Adolescent)) AND ((Autopsy) OR (Pathology) OR (Histopathology) OR (Post-mortem) OR (Post-humous) OR (Necropsy))
Medrxiv	"COVID-19 AND pediatric AND Pathology" and full text or abstract or title "Autopsy"
Biorxiv	(COVID-19 (OR) SARS-COV-2 (OR) PIMS-TS (OR) MIS-C) AND (pediatric (OR) Adolescent) AND (Autopsy (OR) Pathology)

Four independent reviewers (AT, JH, MP, and SC) conducted the initial search and maintained each study’s quality assessment. The studies were conducted and selected using the Rayyan software (Ouzzani et al. 2016). If any unresolved disagreements occurred, two expert reviewers (GSO and AJ) were consulted, and the decision was made based on their expertise and consensus.

The reviewers used the Joanna Briggs Institute’s (JBI) appraisal checklist to measure the general consistency of case series and case reports (Institute JB 2019). If there were any differences between JBI results, it would be discussed until a conclusion could be reached. In order to be included in this systematic review, the case reports and case series needed to fulfil the majority of JBI criteria.

All data within this review were combined using pooled descriptive tests. The data presented in median and range (or interquartile range) and the percentile were converted into mean and standard deviation (Luo et al. 2018). Therefore, all the means and standard deviations will be combined into a single value using the Cochrane method (Higgins and Li 2019).

## Results

**Fig. 1:**
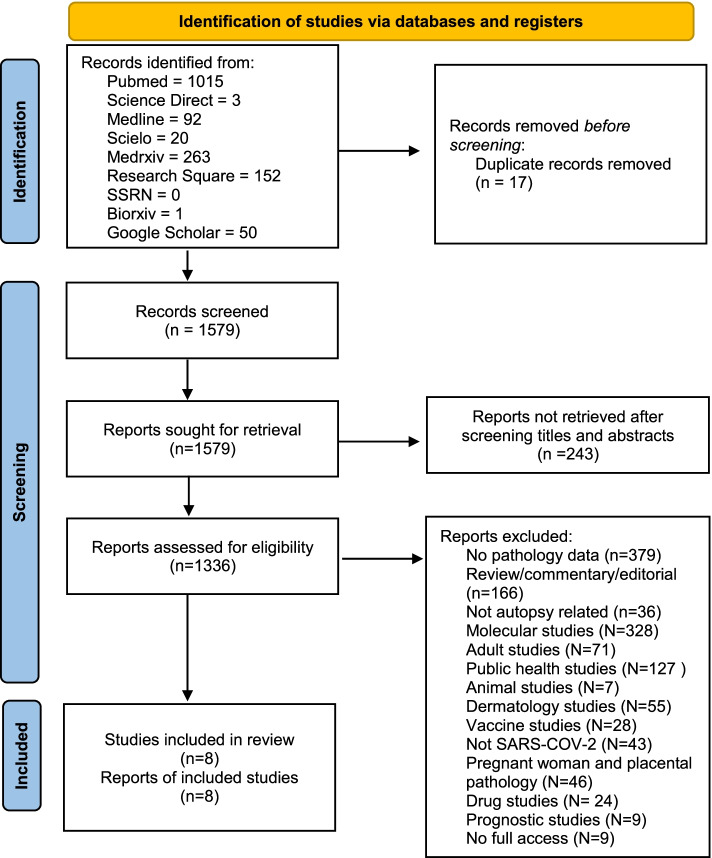
PRISMA flow chart of this study

From the initial search, a total of 1596 articles were identified. After removing duplicates, the authors reviewed 1579 articles for potential relevance based on the titles and abstracts. Subsequently, full texts were reviewed, of which 1571 articles were excluded, leaving eight studies included in this review (Fig. 1). All of the articles were case series (5) and case reports (3). All included studies were rated “good” based on the JBI appraisal checklist. There were 15 patients included in this systematic review, with 77.8% (7/9) being female. The median age of the patients was 11.1 years old (0.6–17 years old) (Table 2). The cause of deaths of patients are specified in only eight patients, ranging from myocarditis (2/8), heart failure (2/8), disseminated thrombosis (1/8), severe COVID-19 pneumonia (1/8), meningoencephalitis (1/8), and colitis (1/8) (Table 3).

**Table 2:** Characteristic of patients demographic

Variables	No of patients available	Median (range)	*N* (%)
Age (years)	15	11.1 (0.6–17)	
Race	8		
Caucasian			2 (25%)
African American			6 (75%)
Sex	9		
Male			2 (22.2%)
Female			7 (77.8%)
BMI	6	22.8 (11.2–31)	
Comorbid diseases	5		
Edwards syndrome			1 (20%)
Diabetes			1 (20%)
Hypertension			1 (20%)
Obese			2 (40%)
First symptom onset (days)	8	10 (2-27)	
**Postmortem COVID-19 testing**			
Antibody	1		
Positive			1 (100%)
Nasopharyngeal PCR	9		
Positive			7 (77.8%)
Lab parameters			Reference range
Hemoglobin, g/dL	7	10.6 (8.8–12.2)	10–15.5
Hematocrit, %	7	31.7 (28.8–39.6)	32–44
Platelets, ×10^3^ cells per μL	7	252.3 (111–296)	150–400
White blood cell count, ×10^3^ cells per mm^3^	7	19 (9.3–32.6)	5–10
Lymphocytes, %	7	11.5 (0.79–58)	20–40
Absolute neutrophil count (K/mm^3^)	6	62.8 (28–95)	55–70
Urea, mg/dL	6	61.3 (23–122)	5–18
Creatinine, mg/dL	6	0.9 (0.26–1.87)	0.5–1.0
D-dimer, ng/mL	6	2532.4 (11.5–2324)	<250
Troponin T, ng/mL	6	0.1 (0.002–0.3)	<0.1
Creatine kinase myocardial band, ng/mL	5	5.76 (0.64–30.7)	0.1–2.88
Creatine kinase, U/L	5	96 (50–1008)	<167
Fibrinogen, mg/dL	5	356 (111–513)	200–393
aPTT, s	4	39.05 (36–57.9)	25.4–38.9
Ferritin, ng/mL	4	707 (159–1501)	20–200
Triglycerides, mg/dL	5	183.5 (59–272)	100–129
Interleukin-6, pg/mL	1	4105	0.2–7.8
Blood pH	1	7.2	7.35–7.45
Bicarbonate, mEq/L	1	15.7	21·0–28·0
PaCO_2_, mm Hg	1	41	35–45
PaO_2_, mm Hg	1	60	80–90
ScvO_2_, %	1	87.2	60–85
Lactate, mg/dL	6	28 (14–39)	4.5–14.4
C-reactive protein, mg/L	7	134.1 (1.5–266.6)	<10
Erythrocyte sedimentation rate (mm/h)	1	74	<10
Total protein, g/dL	2	6.1 (5–7.2)	6–8
Albumin, g/dL	7	3 (2.5–4.3)	3.8–5.4
Total bilirubin, mg/dL	6	3.04 (0.3–11.1)	0.3–1
Direct bilirubin, mg/dL	5	0.37 (0.23–9.84)	<0.2
Aspartate aminotransferase, U/L	7	155.9 (20–506)	10–50
Alanine aminotransferase, U/L	7	142.1 (13–376)	4–36
Gamma-glutamyl transferase, U/L	6	217.5 (9–479)	5–27

**Table 3:** Summary of included papers

Principal investigator (year)	Study cohort (N)/female/(*N*) age (years)^a^/suffered MIS-C	Cause of death	Postmortem NP COVID-19 testing (*N*)	Postmortem assessment (microscopic findings)											
				Heart	Lung	Liver	Kidney	Brain	Bone marrow	Lymph node	Skin	Spleen	Muscle	Colon	Others
Craver et al. (2020)	1/0/17/no	Eosinophilic myocarditis	Positive	Diffuse inflammatory infiltrates composed of lymphocytes, macrophages with prominent eosinophils.	Congestion, acute focal hemorrhage, and edema, but no interstitial inflammation, diffuse alveolar damage, increased intra-alveolar hemosiderin-laden macrophages, viral inclusions, or viral cytopathic changes, eosinophilic infiltrates, vasculitis, or intraparenchymal lymphoid hyperplasia.										
Beaudry et al. (2021)	1/1/18/yes		Positive	Diffuse lymphoplasmacytic inflammatory infiltrates, most marked in the septum. Diffuse lymphoplasmacytic inflammatory infiltrates, most marked in the septum. The inflammation clustered around and partially involved small arterioles, venules, and lymphatics but did not involve capillaries. There were only very focal areas of visible myocyte damage in the right ventricle. Myocardial injury was, in fact, more extensive and occurred too acutely to show diffuse histologic evidence.	Vascular congestion without inflammation, thrombi, or other abnormality.		Focal apoptosis of tubular cells but without frank inflammation, damage, or microthrombi.			Normal.					
Dolhnikoff et al. (2020)	1/1/11/yes	Heart failure		Interstitial and perivascular myocardial inflammation containing lymphocytes, macrophages, a few neutrophils and eosinophils, and foci of cardiomyocyte necrosis; Myocardial necrosis indicated by C4d staining; Myocardial interstitial inflammation containing CD68+ and CD45+ cells.	Pulmonary tissue with focal exudative changes and mild pneumocyte hyperplasia; Fibrinous thrombi in small pulmonary arterioles.	Hepatic centrilobular necrosis	ATN	Reactive microglia		Lymphoid depletion and signs of hemophagocytosis.					
de Almeida Monteiro et al. (2021)	1/n.r./0.6/no			DAD.											
Bhatnagar et al. (2021)	4/n.r./n.r/no		Positive (3), Not performed (1)		Interstitial pneumonitis (1), pulmonary edema (1), DAD, bronchopneumonia (1), tracheobronchitis (1), mild hemorrhage (1).										
Diorio et al. (2020)	1/1/18//no	Heart failure (1)													
Duarte-Neto et al. (2021)	5/4/8.5 (0.6–15)/yes (3)	Disseminated thrombosis (1), COVID-19 pneumonia (1), SARS-COV-2 myocarditis (1), SARS-COV-2 colitis (1), SARS-COV-2 meningoencephalitis (1)	Positive (1), Negative (1), n.r (3)	Interstitial edema (4), pericarditis (1), myocarditis (2), endocarditis (1), myocardial necrosis (3)	Rare cells with cytopathic change (4); Large number of megakaryocytes (2); Typical SARS-COV-2 pneumonia with exudative DAD (3); Thrombi in arterial vessels (3) and septal capillaries (4), Congestion, oedema, foci of haemorrhage (4); Angiomatoid pattern (2); Various foci of coagulative necrosis (1).	Congestion (5), centrilobular necrosis (3), arterial thrombi with ischaemic necrosis (1), sinusoids thrombi (1) syncytial metaplasia of hepatocytes (1), cholestasis (1), hemophagocytosis by Kupffer cells (1), micro/macro vesicular steatosis, multinucleation, mild portal infiltrate (1), hepatocyte binucleation (1)	ATN (5), congestion (5), fibrin thrombi in glomerular capillaries (2), nephrocalcinosis (1), mesangial cell hyperplasia (1), hyaline cast (1), exudate in the Bowman space (1), granular casts (1).	Reactive microglia (5), neuronal ischaemia (5), congestion (5), oedema (2), capillary fibrin thrombi (1), Alzheimer type II glial cells (1).	Hypercellular, hemophagocytosis, emperipolesis by megakaryocytes (1), Normocellular, normomaturative (1); Mild nuclear atypia of megakaryocytes (1).	Pulmonary lymph nodes with lymphoid hypoplasia and hemophagocytosis (1)	Normal (1) Superficial perivascular mononuclear infiltrate (3) Superficial periadnexal mononuclear infiltrate (1)	Splenitis (5), hemorrhages (5), lymphoid hypoplasia with reactive cells (5) hemophagocytosis (2), sinusoidal fibrin thrombi (1)	Myolysis (5), necrotic fibers (4)	Oedema and mild inflammation (1), colitis with dense inflammatory cell infiltration (1), arteriolar microthrombi (1), appendicitis with peritonitis (1)	parotiditis (2), adrenal carcinoma with intense necrosis (1)
Adam et al. (2021)	2/1/11.5 (8–15)/n.r.		Positive (1), Negative (1)		Exudative and proliferative DAD, with epithelial atypia which extended throughout the respiratory epithelium (2).						Vacuolization of the cytoplasm and nucleus (1)				

*ATN* Acute tubular necrosis, *DAD* Diffuse alveolar damage, *N* Number, *n.r* Not reported, *NP* Nasopharyngeal

^a^When there are multiple patients, the data is reported in the median (range)

The most notable laboratory findings for COVID-19 were lymphocytopenia, elevated D-Dimer, increased creatinine kinase myocardial band, increased activated partial thromboplastin time (aPTT), increased ferritin, increased interleukin-6, decreased bicarbonate, decreased lactate, increased CRP, increased (ESR), decreased albumin, increased total bilirubin and direct bilirubin, elevated (AST) and alanine aminotransferase (ALT), and increased gamma-glutamyltransferase (GGT) (Table 3).

Pathology findings from the heart of COVID-19 pediatric patients include diffuse inflammatory infiltrate (2/8), myocarditis (3/8), cardiomyocyte necrosis (4/8), pericarditis (1/8), and interstitial edema (4/8). In one patient, more than one findings are observed, including the results presented in this paragraph and the following ones. Histopathology abnormalities were observed in the lungs of eight patients. These include diffuse alveolar damage (5/8), cytopathic changes (5/8), thrombi in arterioles and septal capillaries (4/8), lung congestion (6/8), focal acute hemorrhage and edema (5/8), focal exudative changes, and mild pneumocyte hyperplasia (1/8). The liver findings of COVID-19 pediatric patients were hepatic congestion (5/6), centrilobular necrosis (4/6), arterial thrombi with ischaemic necrosis (1/6), cholestasis (1/6), sinusoids thrombi (1/6), steatosis (1/6), syncytial metaplasia of hepatocytes (1/6), hemophagocytosis (1/6), portal infiltrate (1/6), and hepatocyte binucleation (1/6).

Histopathological examination of the kidney showed acute tubular necrosis (6/7), congestion (5/7), thrombi in capillaries (2/7), tubular apoptosis (1/7), hyaline and granular casts (1/7), nephrocalcinosis (1/7), and mesangial hyperplasia (1/7). The pathology findings of the brain revealed reactive microglia in all patients (6/6), neuronal ischemia (5/6), congestion, edema (2/6), capillary fibrin thrombi (1/6), and Alzheimer type II glial cells (1/6). The gross neuropathological findings in COVID-19 autopsies were reactive microglia (6/6), neuronal ischemia (5/6), cerebral oedema (5/6), cerebral congestion (1/6), Alzheimer type II glial cells (1/6), and formation of capillary fibrin thrombin (1/6). One patient’s post-mortem bone marrow finding showed hypercellular, emperipolesis and mild nuclear atypia of megakaryocytes (Table 3).

In COVID-19 patients, the lymph nodes showed lymphoid depletion and signs of hemophagocytosis (2/6), lymphoid hypoplasia in pulmonary lymph nodes (1/6), and normal lymph nodes (1/6). The skin biopsy revealed normal histology (1/3), superficial perivascular mononuclear infiltrate (3/3), and periadnexal mononuclear infiltrate (1/3). Pathological findings of the spleen were splenitis (5/5), hemorrhage (5/5), lymphoid hypoplasia with reactive cells (5/5), hemophagocytosis (2/5), and sinusoidal fibrin thrombi (1/5). In a muscle biopsy, myolysis (5/5) and necrotic fibres (4/5). Histopathological findings of the colon showed edema, mild inflammation, colitis, arteriolar microthrombi, and appendicitis with peritonitis. Parotiditis (2/5) and adrenal carcinoma with intense necrosis were also found in biopsies of COVID-19 patients.

Postmortem COVID-19 testing was done with a positive nasopharyngeal test (7/9) and a positive antibody test (1/1). The onset of the first symptoms among patients was ten days (2-27). Among all patients, five patients had comorbidities. The reported comorbid diseases were Edwards syndrome (1), diabetes (1), hypertension (1), and obesity (2). One patient was found to have an congenital immunodefficiency error of immunity. In the performed blood culture, the content of the blood was sterile (1/3), naloxone (1/3), and caffeine positive (1/3). Vitreous examinations were positive for glucose (1) and acetone (1). We found that the weight of the right and left lungs of COVID-19 patients were 955 g (880–1030 g) and 835 g (770–900 grams), respectively. One autopsied patient had a heart weighing 500 g with 80 mL pericardial fluid (Table 4).

**Table 4:** Postmortem examination

Postmortem examination	Number of patients available	Median (range)	*N* (%)
Heart	1		
Weight (grams)		500	
Pericardial fluid (mL)		80	
Lung	2		
Weight (grams)			
Right		955 (880–1030)	
Left		835 (770–900)	
Culture	1		
*Candida glabrata*			1 (100%)
Nasopharyngeal polymerase chain reaction (PCR) test	4		
Positive			3 (75%)
Periodontium			1 (25%)
Heart ultrasound	1		
Thickening			1 (100%)
Heart echocardiogram	5		
Small pericardial effusion			2
Metastatic tumor			1
Patent foramen ovale			1
Apical ventricular septal defect			1
Left ventricular global hypokinesis			1
Dilated left ventricle			1
Right ventricle systolic dysfunction			1
Hyperechogenic coronary arteries			1
Coronary artery diffuse ectasia			1
Left anterior descending coronary artery diffuse ectasia			1
Computed tomography (CT) angiography			
Normal			1 (100%)
Cerebrospinal fluid (CSF) culture			
Contaminated			1 (100%)
Immunodeficiency testing			
Congenital Immunodeffiency			1 (100%)
**Post-mortem toxicology**			
Blood culture	1		
Sterile			1 (100%)
Caffeine	1		
Positive			1 (100%)
Naloxone	1		
Positive			1 (100%)
Vitreous examination			
Glucose	1		
Positive			1 (100%)
Acetone	1		
Positive			1 (100%)

## Discussions

Our study includes eight studies and describes the histopathology or autopsy findings from pediatric COVID-19 patients. Some of the COVID-19 pediatric patients experience prolonged illness duration. A similar result is found in other studies that state the pediatric patient’s median illness duration was 6 days, with 4.4% of the children having an illness duration of at least 28 days (Molteni et al. 2021). Obesity is found to increase the probability of SARS-COV-2 infection in pediatric patients by 39%. Additionally, immunodeficiencies and hypertension are associated with a higher probability of hospital admission with SARS-COV-2 (Leon-Abarca 2020). Patients with diabetes are at increased risk of severe COVID-19 infection, partially due to decreased T cell-mediated immune response (Pozzilli and Leslie 1994).

The majority of the patients in this review experience lymphopenia. One meta-analysis states that patients with poor outcomes had a lower lymphocyte count (Huang and Pranata 2020). This review finds that COVID-19 patients had elevated D-dimer and activated partial thromboplastin time (aPTT) level. It is found that D-dimer and aPTT elevation are associated with an increase in disease severity due to hyperfibrinolysis state and increased inflammatory burden induced in SARS-COV-2 infection (Long et al. 2020). Hyperferritinemia condition has been known to have direct immune-suppressive and pro-inflammatory effects contributing to the cytokine storm (Abbaspour et al. 2014). As a mediator of an inflammatory response, one study finds higher IL-6 levels in patients with complicated COVID-19 associated with higher mortality risk (Grifoni et al. 2020). Decreased bicarbonate and increased lactate levels in the blood indicate an acidosis state in patients with COVID-19 (Nechipurenko et al. 2021).

Studies show that elevated CRP and low albumin levels in children with MIS-C are due to the inflammatory effect (Samprathi and Jayashree 2021). Compared to another study, our study finds similar results that children with severe COVID-19 and MIS-C have low serum albumin levels (Loffredo et al. 2021). Others argue that liver enzymes abnormalities are associated with a condition known as hemophagocytic lymphohistiocytosis (HLH)/macrophage activation syndrome (MAS), which also align with the findings of the current study and may indicate a direct association of the virus with immune dysfunction in severe infection (Duarte-Neto et al. 2021).

Lung congestion and diffuse alveolar damage (DAD) are the most common histopathological manifestation in severe COVID-19 patients. DAD consists of permanent damage to the alveoli epithelial cells and capillary endothelial cells due to phenotypic expression from different proteins transcription modulated by COVID-19 infection (Konopka et al. 2020). Fibrin thrombosis may occur in the proliferative phase of DAD due to neutrophil extracellular traps (NETs) influence (Fuchs et al. 2012). Transmigration of blood proteins as a SARS-COV-2 inflammatory response is also believed to cause focal edema found in this systematic review (Matthay et al. 2019). In comparison to a study of lung autopsies by Zhao et al. (2021), this systematic review has the same results that show pulmonary edema in the lung related to the increased weight of the lung.

The most significant pathologic findings of the heart are cardiomyocyte necrosis, interstitial edema, followed by myocarditis, and inflammatory infiltrate. The direct mechanism of cardiac injury involves SARS-COV 2 that enters human cells by binding its spike protein into the membrane of protein angiotensin-converting enzyme 2 (ACE2) (Hoffmann et al. 2020). ACE2 is found on the ciliated columnar epithelium of the respiratory tract, type II pneumocytes, and cardiomyocytes. Therefore, it is plausible for SARS-COV-2 to infect the human heart, especially with heart failure in which the ACE2 receptor is upregulated (Guo et al. 2020). Moreover, another study proposes that viral myocarditis is caused by T-lymphocyte-mediated cytotoxicity induced cytokine storm syndrome (Esfandiarei and McManus 2008). Interleukin 6 (IL-6) is the central mediator that contributes to the pro-inflammatory response from T-lymphocytes, leading to a positive feedback loop of immune activation and myocardial damage (Esfandiarei and McManus 2008). The severe systemic hyper inflammation and cardiac stress secondary to respiratory failure contribute to the indirect mechanism of cardiac injury (Akhmerov and Marbán 2020). Furthermore, patients included in this review had elevated creatinine kinase myocardial band (CK-MB) levels and heavier heart weight that may indicate cardiac damage, such as viral myocarditis (O’Brien 2008).

Hepatic injuries are also prevalent in children with severe COVID-19. Another study states hepatic congestion was due to liver pressure level (right atrial pressure) in severe COVID-19 patients (Dooki et al. 2020). This review finds that most patients experienced hepatic congestion, followed by centrilobular necrosis. Those findings might occur due to systemic inflammation and shock (Duarte-Neto et al. 2021).

Kidney dysfunction can arise in critically unwell children. In a study of 52 children hospitalized to a tertiary care hospital, 15 (29%) satisfied the British Association of Pediatric Nephrology’s criteria for acute kidney injury (AKI) (Stewart et al. 2020). The majority of AKI cases occurred in children admitted to the intensive care unit (ICU). Our study shows that the common kidney histopathology in children are ATN, congestion, and thrombi formation, with mesangial hyperplasia being the least common. These kidney problems are also apparently due to the MIS-C (Irfan et al. 2021).

The result of our review indicates that the most common neuropathological findings in COVID-19 autopsies are the reactive microglial cells. Microglia is the CNS-resident innate immune cells that cope with invading agents through phagocytosis and the release of pro-inflammatory cytokines (Tremblay et al. 2020). It has been thought that reactive inflammation of microglia is related to a systemic response or secondary to other pathological mechanisms like infarcts or hemorrhages related to coagulation disequilibrium and not by direct consequence of the virus (Maiese et al. 2021). Postmortem analyses of the brain from COVID-19 pediatric patients also show various neuropathological changes of different severity ranging from brain edema, congestion to neuronal loss. It is reported that those changes occurred secondary to hypoxic phenomena (Mukerji and Solomon 2021).

Hemophagocytosis and lymphoid hypoplasia in lymph node autopsy findings may indicate a direct association of the virus with immune dysfunction in severe infection (Duarte-Neto et al. 2021). Other studies that perform skin biopsies found superficial perivascular infiltrate and perivascular mononuclear infiltrate, similar to this systematic review (Amatore et al. 2020). It is believed to be caused by the SARS-COV-2 virus or adverse medication reaction. Hemorrhage and other injuries in spleen findings can be caused by direct damage by the SARS-COV-2 virus that affects macrophages, dendritic cells, pulp cells, and endothelial cells of the blood vessels in the spleen after severe COVID-19 immune dysfunction (Revzin et al. 2020; Schurink et al. 2020).

Myolysis-causing viral infections are more common in children than in adults. Myolysis has recently been identified as one of the complications of COVID-19. Pigmented casts with high creatinine kinase (CK) levels were observed in several COVID-19 patients’ post-mortem renal histopathology analyses in China, presumably indicating myolysis (Su et al. 2020). On abdominal imaging or a colonoscopy, some children with COVID-19 have been found to have terminal ileitis or colitis (Tullie et al. 2020). Our results also show that the COVID-19 may affect this organ system, particularly the colon. This review finds parotitis as the atypical presentation of COVID-19 with one patient who had positive RT-PCR SARS-COV-2 in the parotid gland. Other cases of COVID-19 parotitis have been published in adult patients (Fisher et al. 2021).

There are several limitations in our review. Firstly, our systematic review has a low sample, with most of the studies being case reports which reflect the rarity of pediatric post-mortem findings with COVID-19 infections. Secondly, not all corresponding authors provide the data required in this review (de Almeida Monteiro et al. 2021; Diorio et al. 2020). Moreover, even though pediatric patients were diagnosed with COVID-19 before the autopsy, causal connections regarding the exact cause of death cannot be obtained since it could be related to COVID-19 or other comorbidities (Dewi et al. 2021). However, despite the low samples, this systematic review still gives insights into the autopsies findings in pediatric COVID-19 patients. To our knowledge, this is the first systematic review to elucidate the post-mortem findings from pediatric COVID-19 patients. We highly suggest that more studies have to be conducted to look for the pathophysiology of COVID-19 in the pediatric population. New experimental findings suggest that thrombosis is caused by a soluble adenoviral protein spike variant emerging from splicing events that generate significant endothelial inflammatory events and binding to ACE2-expressing endothelial cells. This process could possibly be linked to severe SARS-CoV-2 infections and pseudovirus infections (Bilotta et al. 2021). However, more research is urgently needed to elucidate the pathophysiology of COVID-19.

## Conclusions

This systematic review describes the histopathology or autopsy findings from pediatric COVID-19 patients. Cardiomyocyte necrosis and interstitial edema are the most significant pathologic findings of the heart. Histopathological examinations of the lungs show lung congestion and diffuse alveolar damage. The autopsy analysis also found pathology findings from other organs such as the liver, kidney, brain, bone marrow, lymph node, skin, spleen, muscle, colon, parotid gland, and adrenal of COVID-19 pediatric patients. Further studies are required to elucidate the pathophysiology of these findings.

## Data Availability

All case-related information is available upon request.

## Abbreviations

COVID-19: Coronavirus disease 2019
MIS-C: Multisystem inflammatory syndrome in children
SARS: Severe acute respiratory syndrome
SARS-CoV-1: SARS coronavirus 1
MERS: Middle East Respiratory Syndrome
PRISMA: Preferred reporting item for systematic review and meta-analysis
PaCO_2_: Partial pressure of carbon dioxide
PaO_2_: Partial pressure of oxygen
ScvO_2_: Central venous oxygen saturation
CRP: C-reactive protein
ESR: Erythrocyte sedimentation rate
AST: Aspartate aminotransferase
ALT: Alanine aminotransferase
GGT: Gamma-glutamyl transferase
JBI: Joanna Briggs Institute’s
aPTT: Activated partial thromboplastin time
HLH: Hemophagocytic lymphohistiocytosis
MAS: Macrophage activation syndrome
DAD: Diffuse alveolar damage
NETs: Neutrophil extracellular traps
ACE2: Angiotensin-converting enzyme 2
IL-6: Interlukin 6
CK-MB: Creatinine kinase myocardial band
AKI: Acute kidney injury
